# Supplementary material for: Scan-Centric, Frequency-Based Method for Characterizing Peaks from Direct Injection Fourier Transform Mass Spectrometry Experiments
Source: Metabolites. 2022 Jun 2;12(6):515. doi: 10.3390/metabo12060515 (PMC9228344; doi:10.3390/metabo12060515)
Supplement: Supplementary file 1 [file metabolites-12-00515-s001.zip › metabolites-1707660-supplementary-v2.pdf]

Bruker SolariX ICR Frequency Conversion

In contrast to the m/z to frequency conversion equations used for Thermo-Fisher Fusion and other Orbitrap instruments, the one we have used for Bruker SolariX ICR instruments is simpler:

$$frequency = a + x \times \frac{1}{mz} + y \times \frac{1}{\sqrt{mz}} \tag{1}$$

Alternative Frequency Models

We can define several models, and test them on the four samples.

$$frequency = a + \frac{y}{\sqrt{mz}} \tag{2}$$

$$frequency = a + \frac{x}{mz} + \frac{y}{\sqrt{mz}} \tag{3}$$

$$frequency = a + \frac{x}{mz} + \frac{y}{\sqrt{mz}} + \frac{z}{\sqrt[3]{mz}} \tag{4}$$

$$frequency = a + \frac{y}{\sqrt{mz}} + \frac{z}{\sqrt[3]{mz}} \tag{5}$$

Figure S1 shows the median absolute deviation (MAD) and medians of the residuals between the calculated and predicted frequency values after fitting of frequency to m/z in each scan using each model, and then across each sample. It is clear that model 3 that includes all three of the m/z terms performs the best in terms of overall fit, although there may be some disagreement between the ECF and lipid samples based on median of the residual differences. Table S1 shows the coefficients for each term of the models for the 1ecf sample. The coefficients of the **y** term in each of the models is orders of magnitude larger than the coefficients for any of the other terms in the other models. That term also appears relatively constant, with some small variations to account for the presence and absence of the other terms of the model.

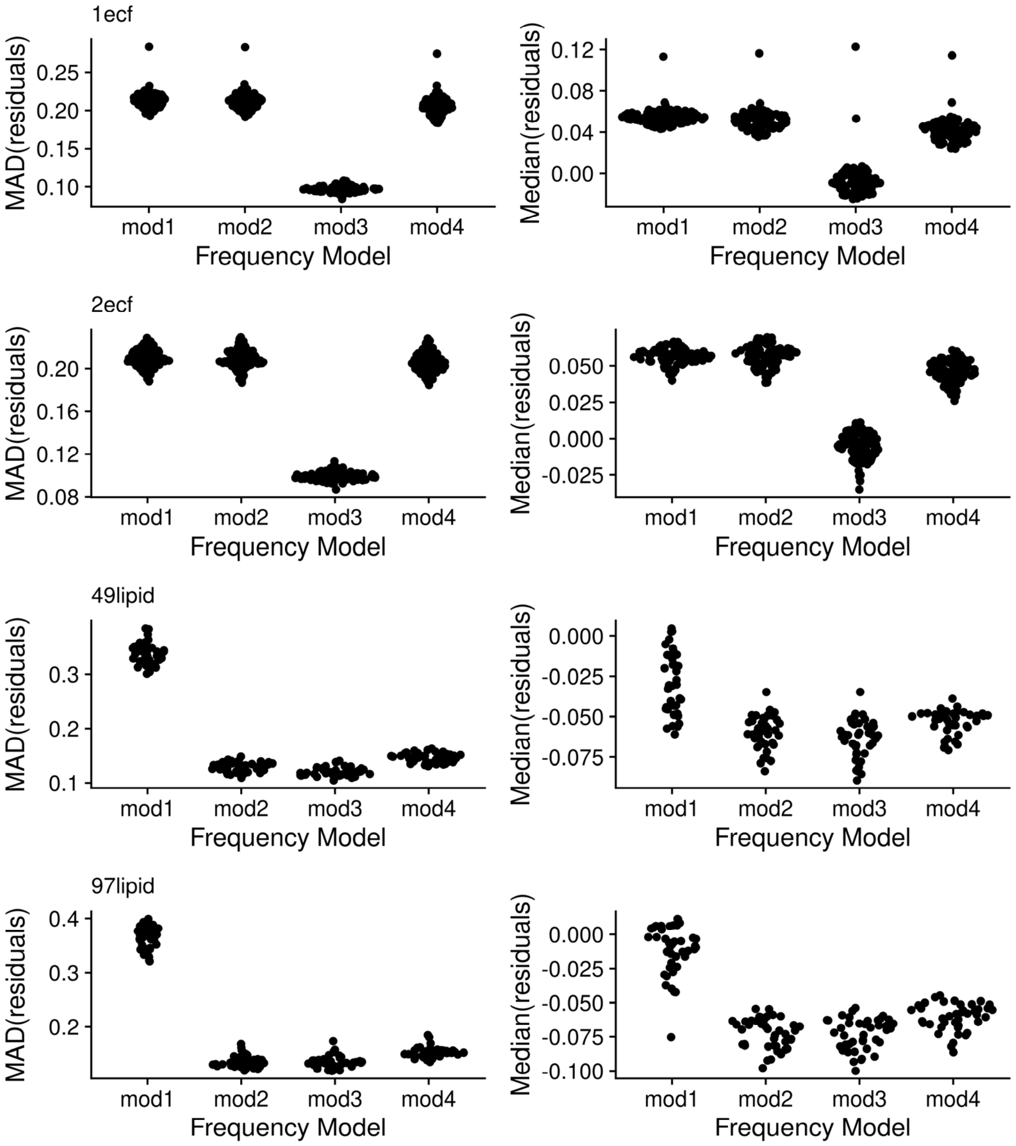

Figure S1. Sina plots of the median absolute deviation (MAD) and median of the residuals between the calculated frequency using the m/z spacing and the predicted frequency after fitting a frequency model, for each of the four samples and for each of the models (1–4) defined above.

Table S1. Coefficients for each term in the models after fitting of the calculated frequency to m/z.

a.freq	x.freq	y.freq	z.freq	model
4		29,803,501		mod1
4	36	29,803,497		mod2
-27	4,508	29,801,327	947	mod3
4		29,803,493	5	mod4

RSD of Methods for Higher Intensity Peaks

Figure S2 and Table S2 we plot and summarize the RSD distributions by sample and method for those peaks with a Log10(mean) intensity ≥ 5. The distributions are shifted to the left compared to using all peaks, but the overall patterns are the same.

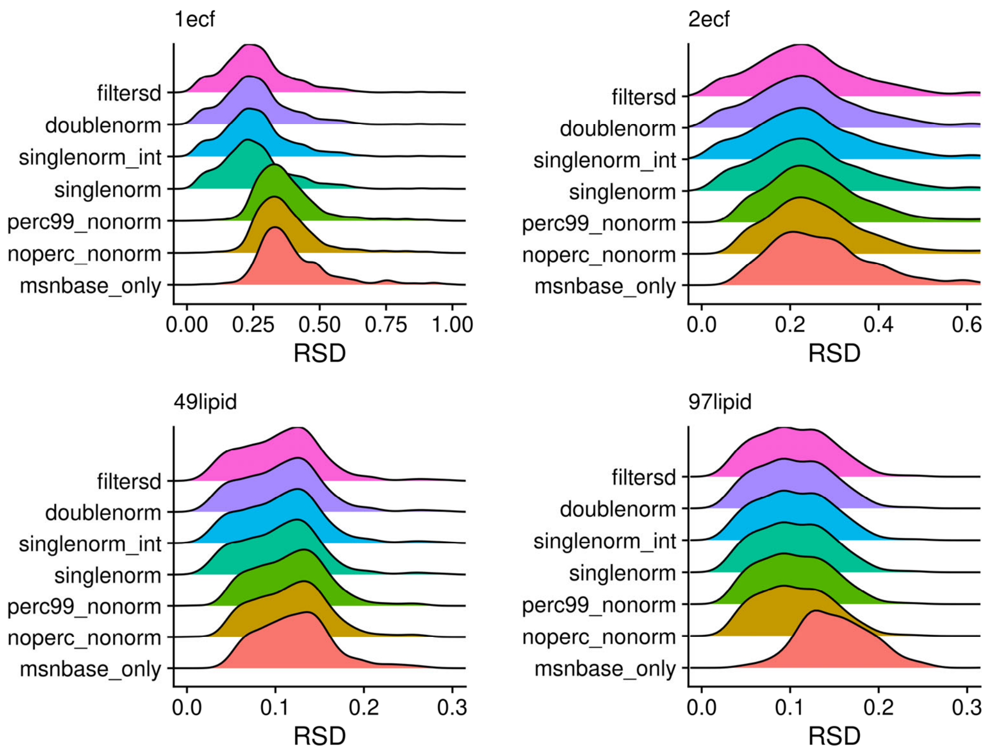

Figure S2. Density plots of relative standard deviations (RSD) of peak heights across scans for each of the processing methods. A peak had to have a Log10(mean) intensity ≥ 5 for the RSD value to be reported.

Table S2. RSD means, medians, modes, and maximum observed values for each sample with different overall processing. Only peaks with a Log10(mean) intensity ≥ 5 are included.

sample	processed	mean	sd	median	mode1	mode2	max
1ecf	filtersd	0.26	0.13	0.25	0.23		1.37
1ecf	doublenorm	0.26	0.13	0.25	0.23		1.37
1ecf	singlenorm_int	0.26	0.14	0.25	0.23		1.41
1ecf	singlenorm	0.26	0.14	0.24	0.23		1.43
1ecf	perc99_nonorm	0.38	0.13	0.35	0.33		1.19
1ecf	noperc_nonorm	0.38	0.13	0.35	0.33		1.19
1ecf	msnbase_only	0.40	0.14	0.36	0.33		1.19
2ecf	filtersd	0.25	0.14	0.23	0.22		0.92
2ecf	doublenorm	0.25	0.14	0.23	0.22		0.92
2ecf	singlenorm_int	0.25	0.14	0.23	0.23		0.92
2ecf	singlenorm	0.25	0.14	0.23	0.23		0.93
2ecf	perc99_nonorm	0.27	0.13	0.25	0.22		1.04
2ecf	noperc_nonorm	0.27	0.13	0.25	0.22		1.04
2ecf	msnbase_only	0.28	0.13	0.26	0.21		0.99
49lipid	filtersd	0.11	0.08	0.11	0.12		1.08
49lipid	doublenorm	0.11	0.08	0.11	0.12		1.08
49lipid	singlenorm_int	0.11	0.08	0.11	0.13		1.08
49lipid	singlenorm	0.11	0.08	0.11	0.13		1.07
49lipid	perc99_nonorm	0.12	0.08	0.12	0.13		1.07
49lipid	noperc_nonorm	0.12	0.08	0.12	0.13		1.07
49lipid	msnbase_only	0.13	0.09	0.12	0.13		1.07
97lipid	filtersd	0.12	0.12	0.10	0.09	0.12	2.05
97lipid	doublenorm	0.12	0.12	0.10	0.09	0.12	2.05

sample	processed	mean	sd	median	mode1	mode2	max
97lipid	singlenorm_int	0.12	0.12	0.10	0.09	0.12	2.05
97lipid	singlenorm	0.12	0.12	0.10	0.09	0.12	2.04
97lipid	perc99_nonorm	0.12	0.12	0.10	0.09		2.03
97lipid	noperc_nonorm	0.12	0.12	0.10	0.09		2.03
97lipid	msnbase_only	0.17	0.15	0.15	0.13		1.94

Peak Variance

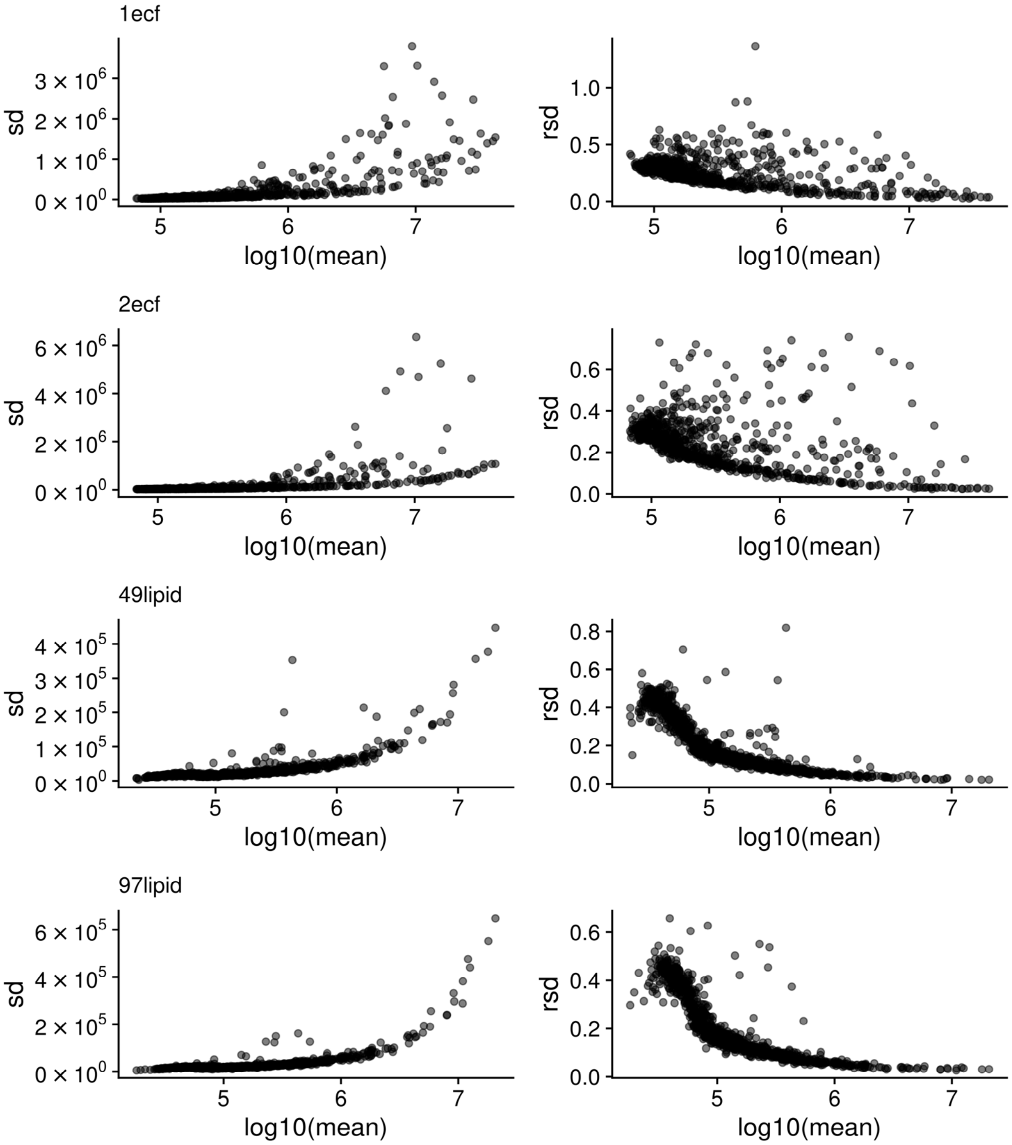

Figure S3. Standard deviation (SD) and relative standard deviation (RSD) as a function of Log10(mean) intensity for peaks present in ≥ 90% of scans for each sample.

Height - NAP Differences of Corrected vs Raw Scan-Centric

Here in Figure S4 we show the differences in Height-NAP peaks using the corrected and raw scan-centric intensities so they can be compared with the differences we observed between Xcalibur and scan-centric peaks.

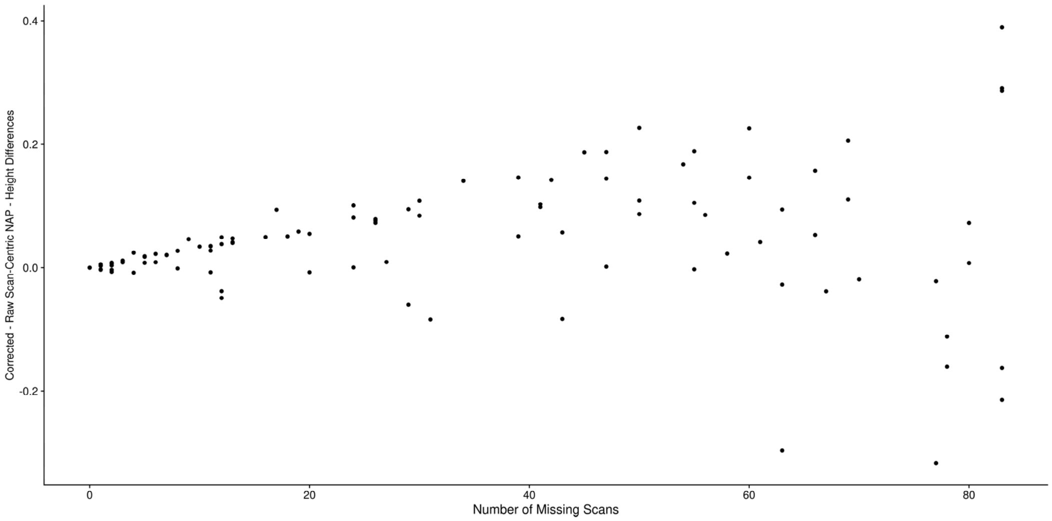

Figure S4. The peak-peak NAP - intensity log differences between corrected and raw scan-centric peak heights as a function of the number of scans the peaks were not found in across all of the amino acid assignments in EMFs with more than a single peak in both ECF samples.

Incorrect Normalization in *P*-Value Differences

During the analysis of the *p*-values between the two classes of NSCLC samples, we didn't originally have the median intensities for the corrected peaks. Therefore, we went ahead and normalized the corrected intensities in each sample with the raw median intensities in each sample, and compared the *p*-values from each method. Figure S5 shows how this incorrect median normalization affects the difference in *p*-values between corrected and raw intensity.

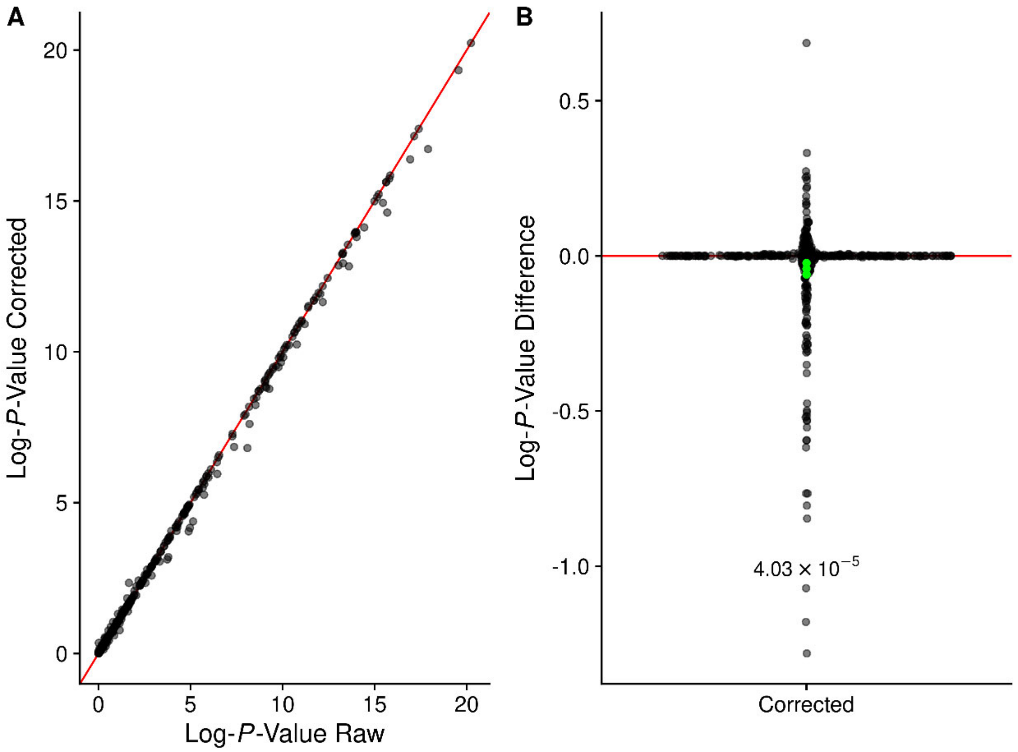

Figure S5. A: Log-*p*-values generated by comparing non-cancer and cancer sample IMFs using peak intensities from raw and corrected peak intensities, where the corrected intensities were normalized using the sample raw median intensities.

Red line denotes perfect agreement. **B**: Sina plot of differences in the log-p-values generated by corrected intensity normalized by the raw median intensities. Also shown are the Bonferroni adjusted p-values from a t-test of the log-p-value differences for each method. Green points denote the high, mean, and low-confidence limits reported from the t-test.

ICI-Kt Median Correlation Outliers

For the NSCLC dataset, we used information-content-informed Kendall-tau (ICI-Kt) correlation within each of non-cancer and cancer sample groups to determine outliers that should be removed prior to statistical testing. In Figure S6 we show the distribution of median ICI-Kt values and the samples determined as outliers.

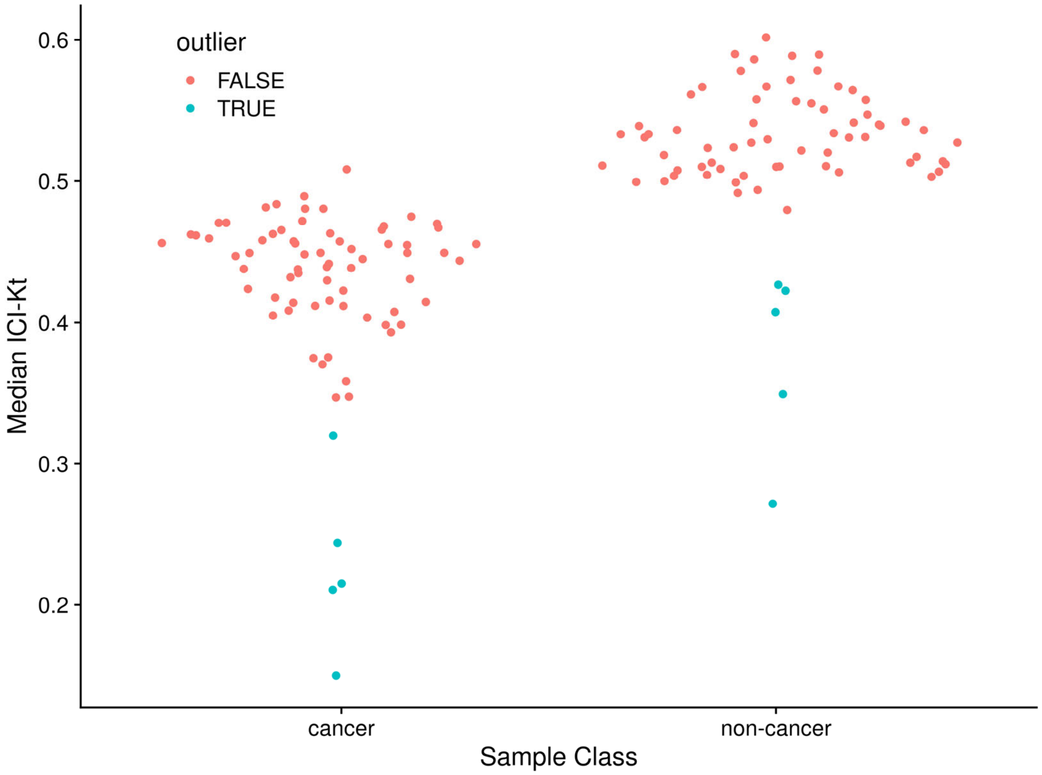

Figure S6. Sina plot of median ICI-Kt correlation for samples within each of the cancer and non-cancer sample groups with outlier status indicated for each sample.
